# Flagellin-Induced Corneal Antimicrobial Peptide Production and Wound Repair Involve a Novel NF-κB–Independent and EGFR-Dependent Pathway

**DOI:** 10.1371/journal.pone.0009351

**Published:** 2010-02-26

**Authors:** Nan Gao, Ashok Kumar, Jeevan Jyot, Fu-Shin Yu

**Affiliations:** 1 Departments of Ophthalmology, Anatomy, and Cell Biology, Wayne State University School of Medicine, Detroit, Michigan, United States of America; 2 Department of Medicine/Infectious Diseases, University of Florida, Gainesville, Florida, United States of America; Johns Hopkins School of Medicine, United States of America

## Abstract

**Background:**

The bacterial protein flagellin plays a major role in stimulating mucosal surface innate immune response to bacterial infection and uniquely induces profound cytoprotection against pathogens, chemicals, and radiation. This study sought to determine signaling pathways responsible for the flagellin-induced inflammatory and cytoprotective effects on human corneal epithelial cells (HCECs).

**Methodology/Principal Findings:**

Flagellin purified from *Pseudomonas aeruginosa* (strain PAK) or live bacteria were used to challenge cultured HCECs. The activation of signaling pathways was assessed with Western blot, and the secretion of cytokine/chemokine and production of antimicrobial peptides (AMPs) were measured with ELISA and dot blot, respectively. Effects of flagellin on wound healing were assessed in cultured porcine corneas. L94A (a site mutation in TLR5 binding region) flagellin and PAK expressing L94A flagellin were unable to stimulate NF-κB activation, but were potent in eliciting EGFR signaling in a TGF-α–related pathway in HCECs. Concomitant with the lack of NF-κB activation, L94A flagellin was ineffective in inducing IL-6 and IL-8 production in HCECs. Surprisingly, the secretion of two inducible AMPs, LL-37 and hBD2, was not affected by L94A mutation. Similar to wild-type flagellin, L94A induced epithelial wound closure in cultured porcine cornea through maintaining EGFR-mediated signaling.

**Conclusions/Significance:**

Our data suggest that inflammatory response mediated by NF-κB can be uncoupled from epithelial innate defense machinery (i.e., AMP expression) and major epithelial proliferation/repair pathways mediated by EGFR, and that flagellin and its derivatives may have broad therapeutic applications in cytoprotection and in controlling infection in the cornea and other mucosal tissues.

## Introduction

*Pseudomonas (P.) aeruginosa*, an opportunistic Gram-negative pathogen, is the leading cause of infectious keratitis associated with contact lens users [1], [2], [3]. Like other flagellated bacteria, the body of *Pseudomonas* flagellum consists of a mass of protofilaments, end-to-end polymer of a single protein, flagellin [4]. The expression of flagella has been considered as a virulence trait providing flagellated bacteria motility toward the targeted hosts. Starting in the late 1990s, flagellin was identified as the soluble mediator that causes inflammation at different tissues such as the intestine [5], airway/lung [6], and cornea [7]. The discovery of Toll-like receptors (TLRs) as receptors recognizing pathogen-associated molecular patterns (PAMPs) has led to a proliferation of interest in innate immunity [8], [9], [10]. Ever increased number of studies revealed that mucosal surface epithelia express multiple TLRs and respond to pathogens by activating signaling pathways identical to those in immune cells, resulting in the production of an array of cytokines and chemokines [11], [12], [13]. Hence, once known only a physical barrier, mucosal epithelia are now recognized as an integrated part of innate immunity [14], [15]. Whether the TLR4-LPS axis is functional in various epithelia remains controversial [16], [17], [18], [19], mounting evidence indicates that flagellin, a TLR5 ligand, is necessary and sufficient to trigger inflammatory responses in different epithelial cells including tracheal columnar [20], airway [21], [22], intestinal (IEC) and colon [23], [24], [25], [26], and corneal epithelia [27]. Gene deletion studies revealed that TLR5 is crucial for *in vivo* epithelial recognition of flagellin that resulted in PMN recruitement to the infected lung [22] and for protecting urinary tract from *E. coli* infection [28], [29]. Thus, TLR5 is a key sensor for epithelial cells to recognize Gram-negative bacteria and to mediate mucosal surface innate immunity.

We recently discovered that flagellin treatment induced cell tolerance/reprogramming in cultured human corneal epithelial cells (HCECs). We observed that prolonged falegllin treatment impaired NF-κB activation, muted pro-inflammatory cytokine production, and augmented expression of antimicrobial molecules [30]. Moreover we also showed that TLR5 activation prior to infection attenuated the development of *P. aeruginosa* keratitis [31]. Interestingly, a recent study revelaed that systematic administration of flagellin protected both mice and monkeys from acute radiation and improved their survival, both prior and after lethal doses of irradiation [32]. Flagellin-induced protection against radiation, chemicals and pathogens in mice was shown to be TLR5 and MyD88-dependent [33]. In an *in vitro* study, flagellin, as well as the TLR2 ligand Pam3Cys, was found to induce a set of non-immune epithelial responses including cell migration, wound repair, proliferation, and survival of epithelial cells [34]. The effects of these TLR ligands on epithelial growth and repair are independent of inflammatory cytokine expression and related to their ability to transactivate epidermal growth factor receptor (EGFR) [34]. EGFR and its associated ligands regulate cellular proliferation, differentiation, survival, migration, and wound repair [35]. Hence, it is of great interest to define the role TLR-NF-κB- and EGFR-mediated cellular functions in epithelial cells in response to flagellin, as it has great potential to become a valuable pharmacologic agent for a variety of infectious and degenerative diseases [36].

In this study, we utilized an *P. aeruginosa* flagellin mutant in the TLR5-binding domain (L94A) to investigate the role of TLR5 binding [37] in the corneal epithelial inflammatory response and wound healing. Our results showed that L94A flagellin failed to activate NF-κB but was active in inducing EGFR signaling, leading to the production of antimicrobial peptides (AMPs) in cultured HCECs and to accelerated epithelial wound closure in cultured porcine corneas.

## Methods

### Reagents and Antibodies

Neutralizing anti-TGF-α monoclonal antibody was purchased from Oncogene Research Products. Anti-phospho-IκB-α, anti-IκB-α, anti-phospho-p38, anti-p38, anti-phospho-JNK, anti-JNK, anti-phospho-AKT and anti-AKT antibodies were purchased from Cell Signaling Technology; anti-phospho-ERK1/2, anti-ERK2, anti-phospho-EGFR, anti-EGFR, and anti-hBD-2 antibodies from SantaCruz Biotechnology; TGF-α neutralizing antibody from Oncogen; anti-LL-37 antibody from PANATecs. N-(3-Chlorophenyl)-6,7-dimethoxy-4-quinazolinamine(Tyrphostin AG1478) and [Glu52]-Diphtheria toxin from Corynebacterium diphtheria (CRM197) were from Sigma-Aldrich.

### Human Corneal Epithelial Cell Culture

Human telomerase-immortalized corneal epithelial (HUCL) cells [38], [39] were maintained in a defined keratinocyte serum-free medium (SFM; Invitrogen Life Technologies, Carlsbad, CA) in a humidified 5% CO_2_ incubator at 37°C. Primary HCECs were isolated from a donor corneas (38 year old male without any known ocular diseases) obtained from the Midwest Eye Banks and also used in some experiments. The epithelial sheet was separated from the underlying stroma after overnight dispase treatment at 4°C. The dissected epithelial sheet was trypsinized and cells collected by centrifugation (500×g, 5 min). HCECs were cultured in T25 flasks coated with fibronectin/collagen. Before treatment, the cells were cultured in growth factor-free and antibiotic-free keratinocyte basic medium (KBM; BioWhittaker, Walkersville, MD) for 16 hours (growth factor starvation). To block EGFR signaling, cells were first treated with AG1478, CRM197, and TGF-α antibody for 30 min and challenged with wild-type PAK flagellin.

### Bacterial Strains and Flagellin

All the bacterial strains used in this study were derived from the wild type (WT) *P. aeruginosa* strain PAK [37]. Bacteria were collected by centrifugation at 7000 *g* at 4°C for 20 minutes and resuspended in 50 mM sodium phosphate buffer (pH 7.0). The bacterial suspensions were either placed on Formvar/Carbon 400 mesh grids (42 µm, Ted Pella) for uranyl acetate staining (0.5%), followed by transmission electron microscope (JEM 1010) examination, or blended to remove the flagellin from bacterial bodies. Flagellins were purified and contaminating lipopolysaccharide (LPS) was removed as described in [27]. Identity of flagellin was confirmed with immunoblot analysis using rabbit anti-PA flagellum B antiserum, kindly provided by Dan Wozniak (the Ohio State University School of Medicine). For direct challenge of HUCL cells, PAK, L94A/PAK, and Q83APAK were shaken in tryptic soy broth (Sigma-Aldrich) at 37°C until absorbance at 600 nm reached O.D. 0.5 (corresponding to 1×10^8^ CFU/ml). The bacterial culture was centrifuged at 6000 *g* for 10 min. Bacteria were resuspended in KBM and then used to challenge the growth factor–starved HCECs at a ratio of 50∶1 (bacteria to cell).

### Western Blot Analyses

Cells challenged with either flagellin or bacteria were lysed with radioimmunoprecipitation assay (RIPA) buffer (150 mm NaCl, 100 mm Tris-HCl [pH 7.5], 1% deoxycholate, 0.1% sodium dodecyl sulfate (SDS), 1% Triton X-100, 50 mm NaF, 100 mm sodium pyrophosphate, and 3.5 mm sodium orthovanadate). A protease inhibitor cocktail (aprotinin, pepstatin A, leupeptin, and antipain, 1 mg/mL each) and 0.1 M phenylmethylsulfonyl fluoride were added to the RIPA buffer (1∶1000 dilution) before use. The protein concentration in cell lysates was determined with the bicinchoninic acid detection assay (MicroBCA; Pierce). Proteins were separated by sodium dodecyl sulfate–polyacrylamide gel electrophoresis (SDS-PAGE) in Tris/glycine/SDS buffer (25 mM Tris, 250 mM glycine, and 0.1% SDS) and electroblotted onto nitrocellulose membranes (0.45-µm pores; Bio-Rad, Hercules, CA). After blocking for 1 hour in Tris-buffered saline/Tween (TBST; 20 mM Tris-HCl, 150 mM NaCl, and 0.5% Tween) containing 5% nonfat milk, the blots were probed with primary antibodies overnight at 4°C. The membranes were washed with 0.05% (vol/vol) Tween 20 in TBS (pH 7.6) and incubated with a 1∶2000 dilution of horseradish peroxidase-conjugated secondary antibodies (Bio-Rad) for 60 minutes at room temperature. Protein bands were visualized by chemiluminescence (Supersignal reagents; Pierce).

### ELISA Measurement of Cytokines

Secretion of TNF-α and IL-8 was determined by ELISA. HCECs were plated at 1×10^6^ cells/well in six-well plates. After growth factor starvation, the cells were challenged for various periods either with flagellin or with live *bacteria* (∼MOI 100) with or without flagellin pretreatment. At the end of the culture period, the media were harvested for measurement of cytokines. The ELISA was performed according to the manufacturer's instructions (R&D Systems, Minneapolis, MN). The amount of cytokines in cultured media was expressed as nanogram per milligram of cell lysate.

### Slot Blot Determination of hBD2 and LL-37

Accumulation of hBD-2 and LL-37 in the culture media was detected by slot blot [40]. Briefly, 100 µl supernatant was applied to a nitrocellulose membrane (0.2 µm; Bio-Rad) by vacuum using a slot-blot apparatus (Bio-Rad). The membrane was fixed by incubating with 10% formalin for 1 hour at room temperature, followed by blocking in Tris-buffered saline (TBS) containing 5% nonfat powdered milk for 1 h at room temperature. The membrane was then incubated overnight at 4°C with rabbit anti-human hBD-2 or LL-37 antibody diluted 1∶1000 in TBS containing 5% nonfat powdered milk, 5% goat serum and 0.05% Tween-20. After washing, the membrane was incubated for 1 h at room temperature with goat anti-rabbit IgG conjugated to horseradish peroxidase diluted 1∶2000 with 5% nonfat powdered milk. Immunoreactivity was visualized with Supersignal reagents (Pierce) using X-ray films. The intensity of each dot on a X-ray film was quantified using Photoshop magic wand tool and histogram analysis and expressed as pixels.

### Porcine Corneal Epithelial Wounding and Organ Culture

Porcine eyes were obtained from a local abattoir, transported to the laboratory on ice in a moist chamber, and processed for corneal culture the same day. Epithelial wounding and corneal organ culture were as described [41]. A 5 mm wound was allowed to heal for 48 hr in MEM in the presence or absence of PAK flagellin (250 ng/ml), L94A flagellin (250 ng/mL), with or without AG48098 (1 µM, an EGFR inhibitor), in a humidified 5% CO_2_ incubator at 37°C. At the end of culture, the corneas were stained with Richardson's staining solution to mark the remaining wound area, photographed, and the remaining wound area was quantified using Photoshop software. At least four corneas were used for each treatment and two independent experiments were performed (eight or more corneas for each treatment).

### Statistical Analysis

All values are expressed as the mean ± SD. Statistical analysis was performed with analysis of variance (ANOVA) to determine statistical significance for data from cytokine ELISA and AMP dot blot. Mean differences were considered significant at a *P* value of <0.05.

## Results

### TLR5 Binding Is Required for Flagellin-Mediated Inflammatory Response, but Not for EGFR Transactivation and the Production of Antimicrobial Peptides in HCECs

**Figure 1:**
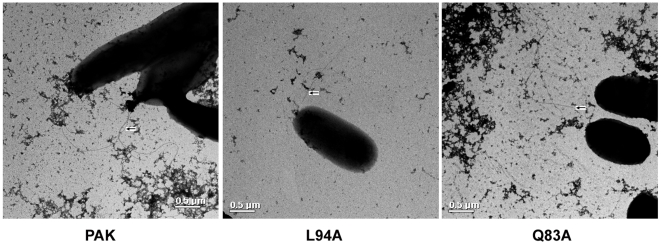
Electron micrographs of wild-type PAK and mutant L94A and Q83. Bacterial were grown to OD600 = 0.5 were centrifugated at 7000 *g* and resuspended in PBS. A drop of bacterial suspensions was allowed to adhere to a carbon-coated grid for 10 s and drained off. Adherent cells were stained with a 0.5% uranyl acetate and examined TEM. Note the presence of flagella on the wild type and mutant bacteria. Scale bar  = 500 Å.

**Figure 2:**
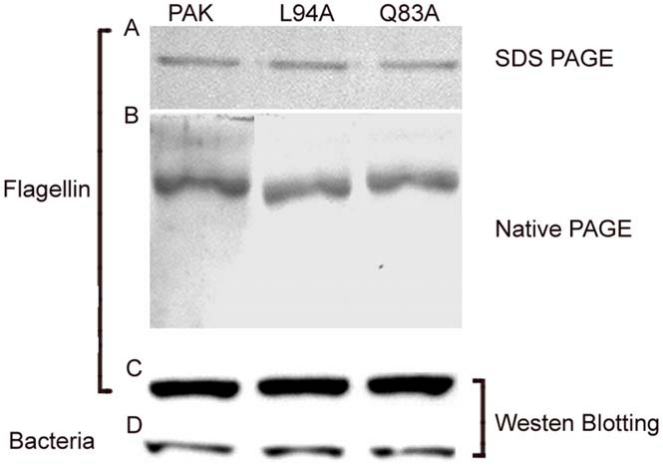
PAGE and immunoblot analysis of PAK flagellins. The purified flagellins (120 ng) were resolved either on 5% to 15% SDS (A) or non-denturing polyacrylamide gels (B) and stained with Coomassie blue. Purifed flagellin (50 ng, C) or bacterial extracts (60 µg, D) resolved on SDS-PAGE and transferred to nitrocellulose were also probed with flagellin antibody.

We previously showed that flagellin purified from PA01 strain stimulated an inflammatory response of HCECs through TLR5 [27]. In this study, we took advantage of flagellin mutants generated from PAK strains. Mutation in the amino acid residue 94 (L→A) that is within a conserved region of flagellin among different flagellated bacteria, but not in 83 (Q→A) outside the binding region, was shown to disrupt TLR5 binding *in vitro* [37]. The flagellin preparation involves centrifugation and suspension of the bacteria, which may result in shearing of the flagellae. To verify whether bacteria retained their flagellae, we performed transmission electron microscopy (TEM) and observed that the wild-type PAK and mutant bacteria (L94A and Q83A) possessed flagella (Fig. 1). Flagellins were purified from these strains with LPS removal (Fig. 2). SDS-PAGE-Coomassie blue staining revealed a predominant band at approximately 60 kDa, consistent with the known molecular weight of *P. aeruginosa* flagellin (Fig. 2A). The monomer nature of prepared flagellins was erified by gel filtration chromatography using FPLC Superdex 200 HR10/30 column showing single peak around 15 ml elusion volume (data not shown) and by non-denaturing PAGE analysis showing a single band (Fig. 2B). Furthermore, immunoblotting with anti-*P. aeruginosa* flagellin antibody detected a single immunoreactive band coincident in molecular weight with the Coomassie blue-stained band indicating that the detected proteins, including purified protein (Fig. 2C) and bacterial extract (Fig. 2D) and, are *P. aeruginosa* flagellin.

**Figure 3:**
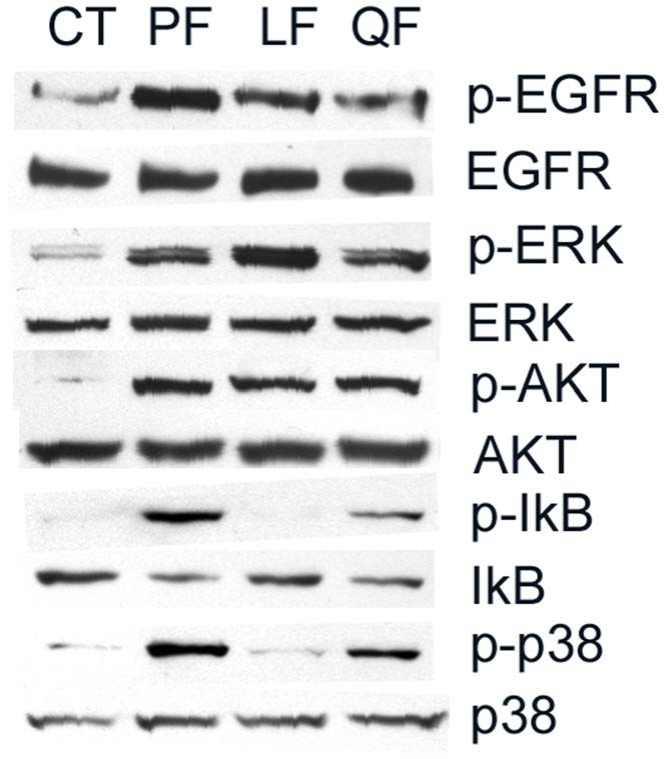
Mutation in TLR5 binding region disturbs NF-κB, but not EGFR-mediated signaling pathways in HCECs. Growth factor-starved primary HCECs cells were either left unchallenged (CT) or challenged with purified flagellin (250 ng/ml) from indicated bacterial strains; PF(wild type PAK), LF (L94A), QF (Q83A), and then lysed 60 min post-stimulation. Equal amounts of proteins of 30–45 µg were subjected to Western blot using antibodies against phospho- (p) proteins IκB-α, P38, EGFR Y^1173^, ERK or AKT. After stripping off the immunoreactivities, the membranes were reprobed with antibodies against, corresponding proteins for proper protein loading. The Figure is a representative of three independent experiments.

Mutation in the amino acid residue 94 (L→A) that is within a conserved region of flagellin among different flagellated bacteria, but not in 83 (Q→A) outside the binding region, was shown to disrupt TLR5 binding *in vitro* [37]. To determine the effects of the L94A mutation on intracellular signaling of HCECs, we treated primary HCECs with purified flagellins and assessed IκB-α and p38 phosphorylation, and IκB-α degradation by Western blotting and the results were shown in Figure 3 (similar results were also obtained using HUCL cells, data not shown). Flagellin isolated from PAK or Q83A (Q) activated the NF-κB and p38 pathways as evidenced by the appearance of Phospho-IκB-α and phospo-p38 in primary cultred HCECs cells 60 min post stimulation. Consistent with an increase in IκB phosphorylation, the level of IκB was also decreased while p38 remained unchanged in the treated cells. No detectable increase in the levels of phospho-IκB-α and phospho-p38 or decrease in IκB-α in cells treated with L94A (L) flagellin were observed.

In addition to activating NF-κB, ligation of a TLR with its ligand has also been shown to activate EGFR in epithelial cells through EGFR transactivation [34], [42], [43]. To determine if EGFR transactivation requires TLR binding, the activation of EGFR and its downstream pathways were also assessed. Western blot analysis revealed that L94A induced EGFR phosphorylation as well as the phosphorylation of ERK and AKT (substrate of PI3K) in a manner similar to that of wild type and Q83A (Fig. 3), suggesting that flagellin-induced EGFR activation may be uncoupled from NF-κB and other inflammatory pathways in HCECs.

### TGF-α Is an Endogenous Mediator for Flagellin-Induced EGFR Transactivation

**Figure 4:**
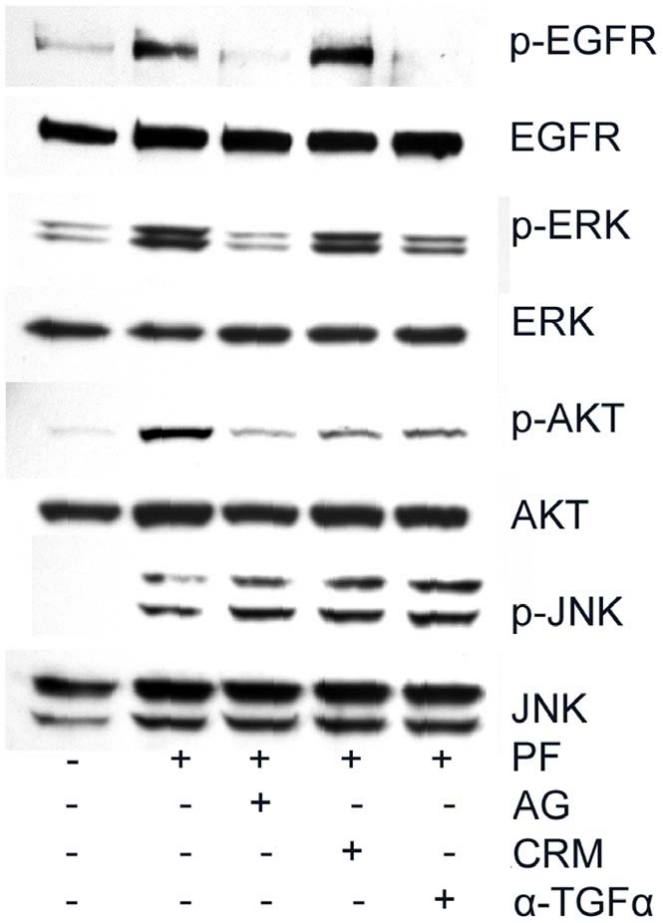
Flagellin induced EGFR activation involves TGF-α and HB-EGF in HCECs. Growth factor-starved HUCL cells were pretreated with AG1478 (AG, 1 µM), CRM197 (CRM, 10 ng/µl), or TGF-α neutralizing antibody (α-TGF-α 5 µg/ml) for 1 h and then challenged with purified wild type PAK flagellin (PF). One hour post flagellin challenge, cells were lysed and equal amounts of proteins of 30–45 µg were subjected to Western blot using antibodies against phospho- (p) proteins JNK, EGFR at Y^1173^, ERK or AKT. After stripping off the immunoreactivities, the membranes were reprobed with antibodies against proteins for proper loading. The Figure is a representative of two independent experiments.

Two common endogenous mediators for EGFR transactivation are heprine-binding (HB)-EGF and TGF-α [44]. Our previous studies showed that wounding and GPCR ligands, ATP and lysophosphatidic acid (LPA), primarily utilize the release of HB-EGF as a means to transactivate EGFR [45], [46]. Having shown that flagellin stimulates EGFR transactivation, we next investigated which of these two EGFR ligands expressed in HCECs are involved in flagellin-mediated EGFR transactivation using HB-EGF blocking agent CRM192 and TGF-α neutralizing antibody. As shown in Figure 4, while AG1489 (the EGFR inhibitor) and to a lesser extent, TGF-α neutralizing antibody and CRM197 (an HB-EGF inhibitor), attenuated EGFR, ERK and AKT phosphorylatione. Thus, unlike GPCR ligands, TLR ligands may depend on both TGF-α and HB-EGF as intermediate mediators for EGFR transactivation. All three inhibitors exhibited no effects on JNK phosphorylation, suggesting that this inflammatory pathway may be parallel to that of the EGFR signaling pathway after flagellin stimulation.

### L94A Mutation Attenuates the Flagellin-Induced Production of Inflammatory Cytokines, but Not Antimicrobial Peptides in HCECs

**Figure 5:**
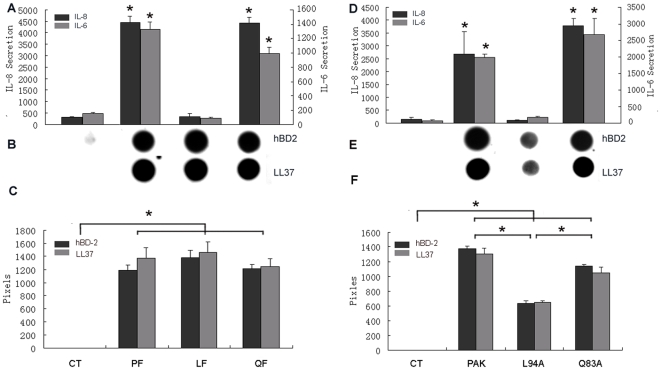
Mutation in TLR5 binding region inhibits flagellin- or live PAK-induced IL-6, IL-8, but not hBD2 and LL-37, production in HCECs. HUCL cells were challenged with either 250 ng/ml flagellin (A–C) purified wild-type (PF) PAK, PAK/L94A (LF), and PAK/Q83A (QF) or live bacteria (D-F) for 4 hr. Culture supernatants were analyzed for IL-6, IL-8 by ELISA (A and D) or for hBD2 and LL-37 production using slot-blot (B and E). The intensity of each dots was determined by Photoshop histogram analysis and expressed mean of pixels (C and F). Data shown are representative of two independent experiments. *P<0.01 (N = 3).

To test whether TLR5 binding is required for flagellin-induced cytokine production, the secretion of IL-6 and IL-8 into the culture media of HCECs was measured (Fig. 5). Similar to purified PA01 flagellin [27], incubation of cultured HUCL cells with PAK flagellin for 4 h resulted in a significant increase of both IL-6 and IL-8 levels. Q83A was similarly effective in induction of IL-8 production, albeit to a less extent for IL-6. No increases in IL-6 and IL-8 secretion were observed in cells treated with L94A (Fig. 5A), consistent with its inability to activate NF-κB in these cells (Fig. 3). Similar pattern of cytokine production was also observed when HUCL cells were challenged with live bacteria, PAK, PAK/L94A, and PAK/Q83A. These bacteria were subjected to centrifugations and re-suspensions and were shown to possess sizable flagella (Fig. 1). While the wild type PAK and PAK/Q83A were simulative, PAK/L94A was ineffective in stimulating IL-6 and IL-8 production (Fig. 5D). This is consistent with ours and others' studies suggesting that for mucosal epithelial cells TLR5 is a major PRR for Gram-negative bacteria including *P. aeruginosa*.

Antimicrobial peptides, particularly β-defensins and LL-37 (a product of human cathelicidin), are important components of the innate defense machinery at mucosal surfaces and the expression of two AMPs, human β-defensin 2 (hBD2) and LL-37, are induced in response to infection in a variety of epithelial cells in a TLR-dependent manner [47], [48], [49], [50]. The media from flagellin or live bacteria challenged cells were subjected to the immunodetection of LL-37and hBD2 (Fig. 5B & E). All three types of flagellins (Fig. 5B & C) and live bacteria (Fig. 5E & F) induced the accumulation of large amount of LL-37 and hBD2, which were not detected in unstimulated cellsdot blot. Interestingly, live L94A, but not L94A flagellin, was significantly less effective in induction of these AMP secretion. HB-EGF, a natural agonist of EGFR, also induced the accumulation of LL-37, but not hBD2 (data not shown).

### TLR5 Binding and NF-κB Activation Are Required for the Flagellin-Induced Tolerance/Reprogramming in HCECs in Response to PAK Challenge

**Figure 6:**
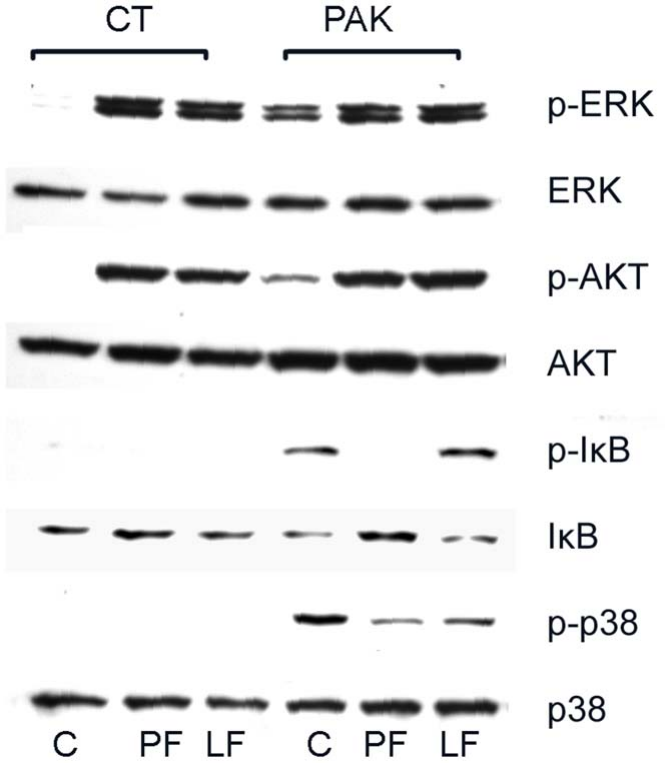
Flagellin Mutated in TLR5 binding region fails to silence PAK-triggered inflammatory signaling pathways in HCECs. HUCL cells were either pretreated with 50 ng/ml PAK (PF) or L94A (LF) flagellin or left untreated (C) for 24 h and then challenged with (PAK) or without (Control) live PAK for 1 h. Total cell lysates were blotted with antibodies specific for phospho-ERK, AKT, IκB-α, and P38, followed by reprobing with antibodies against corresponding proteins. The data shown are representative of 2 independent experiments.

We previously showed that prior exposure of HCECs to flagellin results in the tolerance or reprogramming in HCECs, as manifested by impaired NF-κB activation, muted pro-inflammatory cytokine production, and augmented expression of antimicrobial molecules in response to live bacteria challenge in cultured HCECs [31]. Having shown that L94A was unable to stimulate NF-κB activation, but was functional in triggering EGFR mediated signaling, we next investigated whether this TLR5 binding mutant induces HCEC tolerance in response to PAK challenge and the results are shown in Figure 6. Pretreatment of cells with relatively a low dosage of PAK or L94A flagellin (50 ng/ml) for 24 h increased the phosphorylation of ERK and AKT, indicative of EGFR activation, but not of IκB or p38. Thus, presence of flagellin appears to elicited EGFR activation. Live PAK challenge induced the phosphorylation of IκB, p38, as well as IκB degradation in the control, untreated cells, all of which were blocked by pretreatment with wild type PAK -flagellin. L94A flagellin pre-treatment had no detectable or minimal effects on PAK-stimulated activation of NF-κB and p38.

**Figure 7:**
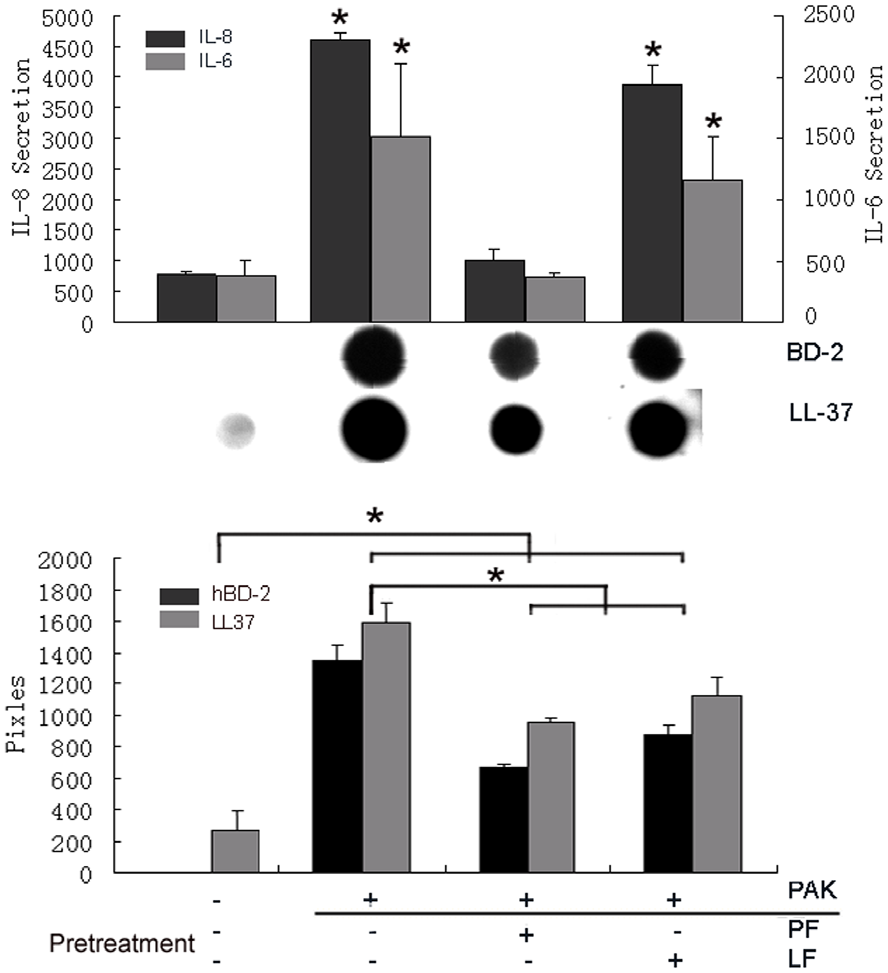
Flagellin Mutated in TLR5 binding region fails to blunt PAK-induced cytokine production in HCECs. HUCL cells were pretreated without (C) or with 50 ng/ml PAK (PF) or L94A (LF) flagellin for 24 h. After washed twice with PBS, cells were stimulated with live PAK for 4 h. Culture supernatant was analyzed for IL-6, IL-8 with ELISA or for hBD2 and LL-37 production using slot-blot. The intensity of each dots was determined by Photoshop histogram analysis and expressed mean of pixels. Data shown are representative of two independent experiments. *P<0.01 (N = 3).

Using PAK as the stimulus, we assessed flagellin and L94A-induced reprogramming by measuring cytokine and AMP productions in cultured HUCL cells (Fig. 7). While pretreatment with low dosage of wild type PAK flagellin inhibited live bacteria induced secretion of IL-6 and IL-8, pretreatment of cells with L94A flagellin exhibited minimal effects. On the other hand, in pretreated cells PAK was still able to induce signiciant accumulation of hBD2 and LL-37 and pretreatment of L94A flagellin had similar effects on their secretion to that of widl type. As 50 ng/ml flagellin was known to induce transient NF-κB activation [31], we conclude that TLR5 binding and/or NF-κB activation are required for flagellin induced HCEC tolerance/reprogramming in CECs *in vitro*.

### Flagellin Promotes Epithelial Wound Closure in a TLR5 Binding- and NF-κB-Independent Manner

**Figure 8:**
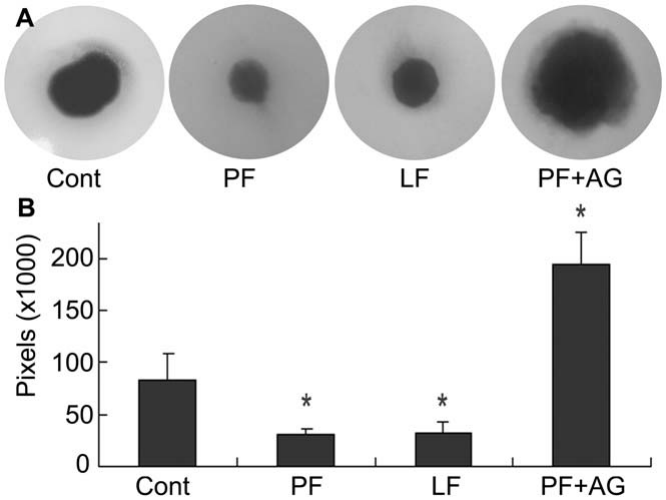
L94A mutation does not affect flagellin-induced epithelial wound closure in cultured porcine corneas. Corneal epithelial wound of 5-mm diameter (the circle representing the original wound size) was made and allowed to heal for 48 h in MEM (control), MEM containing 250 ng/ml PAK (PF) or L94A (LF) flagellin, or 250 ng/ml PAK (PF) plus 1 µM AG1478 (PF+AG). Wounded corneas were stained with Richardson solution to show the remaining wound area (blue) at the end of the culture. (A) Representatives of 5 corneas per condition from 3 independent experiments. (B) Changes in the mean of the remaining wound areas in pixels were calculated from five corneas per condition by histogram of Adobe Photoshop software (*p<0.01).

Having shown that L94A flagellin transactivated EGFR and its downstream signaling pathways but was unable to stimulate cytokine production as wild type and Q83A strains, we then investigated the effects of flagellin mediated EGFR activation on corneal epithelial wound healing using an ex vivo model of a porcine corneal organ culture (Fig. 8). As we showed previously, a wound can be healed spontaneously through autocrine production of EGFR ligands in response to epithelial damage [41]; and presence of wild type PAK flagellin significantly accelerated wound closure in the cultured pig corneas. L94A also accelerated the wound closure in a similar fashion. The accelerating effect of flagellin on epithelial wound closure required EGFR, as it was sensitive to AG1478, an EGFR kinase inhibitor.

**Figure 9:**
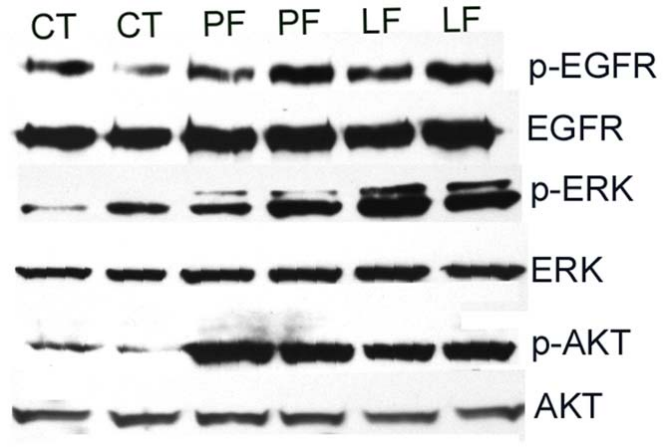
Flagellin upregulates EGFR signaling pathways in the epithelia of healing porcine corneas. Epithelial cells in healing porcine corneas treated with wild type (PF), L94A (LF) flagellin, or medium alone (CT), were collected 48 hrs post wounding, lysed in RIPA buffer and sonicated. Equal amounts of cell lysates from 2 of 5 corneas (randomly picked) from each group were immunoblotted with antibodies against phospho-EGFR, AKT or ERK1/2. The immunoreactivities were stripped off and the same membranes were reprobed with antibodies against cellular EGFR, AKT or ERK2, respectively, for proper protein loading.

To elucidate the mechanism underlying flagellin accelerated wound closure, we next investigated the effects of flagellin on EGFR signaling in cultured corneas (Fig. 9). There were detectable levels of phosphorylation of EGFR, ERK and AKT in healing porcine CECs and the presence of both PAK and L94A flagellin increased the levels of phospho-EGFR, phospho-ERK, and phospho-AKT while the total cellular levels of the proteins remained largely the same in samples tested. The phoshphorylation of IκB or p38 was not detectable in these samples (data not shown).

## Discussion

The ocular surface is constantly exposed to a variety of opportunist pathogens including *P. aeruginosa*. The epithelium forms a physical barrier at the apical layer preventing microorganisms from entering the cellular layers that express pathogen pattern recognizing receptors such as TLR5 [28]. Thus, in the cornea, TLR5-flagellin interaction only occurs when there is an epithelial injury or defect and when there are invading pathogens. In this study, we showed that flagellin mutated at TLR5 binding site failed to induce inflammatory responses that include NF-κB activation and inflammatory cytokine production but maintained the ability to induce the production of AMPs and to promote epithelial wound closure, likely in an EGFR dependent manner. Hence, our data shows that the TLRs may function not only as immune-stimulator to induce the innate response but also as receptors for damage-associated molecular patterns to promote wound repair and reformation of the epithelial bio-defense barrier, including the production of AMPs.

While TLR-mediated inflammatory response via NF-κB activation and other inflammatory pathways is well-studied, the molecular events leading to and the consequence of EGFR activation in epithelial cells are still elusive. As the first line of innate defense, epithelia in a variety of tissues express most, if not all, the 10 human TLRs. These TLRs mediate two distinct signaling pathways, MyD88 dependent (TLR2, 4, 5, 7/8, and 9) and MyD88 independent (TLR3 and 4), leading to cytokine production and inflammatory responses. Yet, the ligands for both MyD88-dependent (TLR2, 4, and 5) and -independent TLRs (TLR3 and 4) have been shown to transactivate EGFR [34], [43], [51]. The activation of EGFR by TLR ligands is generally believed to be a downstream event of TLR-ligand interaction. In this study, we took advantages of TLR5 binding mutations generated form PAK [37] to delineate TLR5 mediated signaling pathways in cultured HCECs. In humans, TLR5 is the only cell surface PRR which recognizes bacterial flagellin. Using the WT and mutated flagellins, we showed that while the wild-type and Q83A flagellins were able to stimulate both the well documented TLR5-MyD88 mediated inflammatory signaling pathways (NF-κB, p38 and JNK) and EGFR signaling pathways, flagellin from L94A strain only activated EGFR and its downstream signaling pathways. Recent studies revealed that flagellin and whole flagella possess different immunological activity [52], [53]. Flagellin was shown as a potent activator of TLR5 and inducing innate immunity whereas flagella induced strong adaptive immunity with generation of antibodies [53]. This may be due to the fact that the TLR5-binding domain of flagellin is not exposed in the flagellum [54]. Hence, it is important to show that that the loss of NF-κB stimulating activity in L94A is not due to unequal ability to form flagellum-like superstructures. The analyses of gel filtration chromatography showing no proteins in exclusion volume and non-denaturing PAGE analysis shwoing one band with similar intensity of Coomassie blue staining for all three flagellin preparations suggest that the proteins used to stimulate HCECs are mostly in monomer forms. Taken together, our results suggest that EGFR and NF-κB pathways can be uncoupled and that flagellin-elicited EGFR activation may require either flagellin-TLR interaction with less affinity or through different and yet unidentified/proved cell surface receptor such as GM1 [55], [56], [57]. Further study to identify flagellin receptor(s) that leads to EGFR transactivation is warranted.

Regardless of mutation sites, flagellin is effective in triggering EGFR transactivation, a process known to be dependent on metalloproteinase-mediated ectodomain shedding of its ligands [44], [58]. The release of EGFR ligands is important in many cellular signaling pathways and is disregulated in many diseases [59]. Our results showed that flagellin-elicited EGFR activation is dependent on both TGF-α and HB-EGF, two well-characterized EGFR ligands involved in its transactivation. Importantly, GPCRs for ATP and LPA as well wounding, require HB-EGF for EGFR transactivation in the same cells [41], [45], [46]. We have shown that for wounding, probably through the release of ATP, rapid and transient Erk activation is involved in initiating EGFR signaling pathways in surrounding, uninjured cells [45], [60]. Hence, TLR ligands such as flagellin may utilize a different intracellular mediator/pathway, such as the generated intracellular reactive oxygen species [51], to induce the release of TGF-α, in addition to commonly used HB-EGF. It is well documented that, whereas HB-EGF binding leads to lysosomal degradation of EGFR, TGF-α causes receptor recycling [61]. Thus, flagellin, through induction of TGF-α release, may stimulate continuous signaling and therefore, be a potent mitogen and/or chemoattractant for HCECs. This is consistent with our results that presence of flagellin in culture media of porcine corneas resulted in a persistent elevation of EGFR signaling (detectable at 48 h), leading to enhanced epithelial wound closure.

Both purified protein and live L94A were unable to stimulate HCEC to produce inflammatory cytokines *in vitro*, consistent with the fact that the L94A mutant failed to induce NF-κB activation. In addition to functional analyses [37], the TEM analysis revealed fully formed multiple flagella in all three strains used, suggesting that the lack of stimulative activity for L94A is not due to the loss of flagella which might be sheared by centrifugation and/or resuspension. In macrophages, both L94A and Q83A failed to stimulate caspase-1 activation and IL-1β secretion, a process depending on Ipaf, an intracellular receptor for flagellin [62]. Ligation of lpaf induces IL-1beta secretion through caspase-1 activation [63]. We recently found that challenging HCECs with either purified flagellin or invasive strains of *P. aeruginosa* resulted in an up-regulation IL-1β at both mRNA and protein levels but not in the secretion of IL-1β (Yu et al unpublished results), suggesting that either flagellin cannot be internalized or Ipaf-caspase axis is not functional in HCECs. Thus, TLR5 is the major pathogen pattern recognizing receptor of HCECs to initiate the inflammatory response toward *P. aeruginosa*. Interestingly, L94A flagellin stimulated EGFR and its downstream signaling pathways, suggesting that TLR5-flagellin interaction, at least the TLR mediated-activation of NF-κB, may not be required for EGFR transactivation. Surprisingly, the expression of two infection-inducible antimicrobial peptides, LL-37 and hBD2, were induced by both purified L94A flagellin and live L94A flagellin at levels comparable to that induced by wild type and Q83A flagellin. L94A was reported to upregulate the expression of surfactant protein D (SP-D) but not IL-8, in another HCEC cell line [64]. Thus, the flagellin-induced production of innate defense molecules may be separated from the inflammatory response, providing a means for the upregulation of protective factors, such as SP-D, LL-37 and defensins, without stimulating proinflammatory response. Moreover, the activation of EGFR may be part of the mechanisms for regulating AMP expression at the ocular surface. Thus, by targeting specific pathways after TLR activation, it is possible to enhance innate defense without the induction of inflammation, hence avoiding its associated tissue damage.

In our previous study to delineate the underlying mechanisms that modulate the inflammatory response after TLR stimulation, we showed that prior exposure of HCECs to flagellin results in tolerance and cell reprogramming in HCECs [30]. Using the production of cytokines and AMPs as parameter, here we showed that mutant L94A flagellin was unable to induce HCEC reprogramming. Hence, we conclude that the initial activation of NF-κB by flagellin is important for the induced cell tolerance/reprogramming.

Recently, several studies revealed that flagellin can evoke a whole panel of cytoprotective responses against chemicals, pathogens, and radiation. Burdelya and her colleagues reported that flagellin or its derivative CBLB502, injected 45 minutes to 24 hours before exposure protected mice and rhesus monkeys from lethal radiation [32]. Systemic treatment of mice with flagellin purified from *Salmonella typhimurium* also protected mice against chemicals and pathogens, in addition to ionizing radiation [33]. Infection and the host inflammatory response usually cause tissue injury and, therefore, rapid healing is essential for restoring and maintaining the tissue integrity and function post-infection. Hence, tissue repair is an important part of innate defense mechanisms at the mucosal surfaces and the underlying mechanisms for tissue repair and cytoportection may be the same. Using corneal organ culture, we showed in the current study that flagellin, with or without TLR5 binding mutation, greatly accelerated *ex vivo* epithelial wound closure in an EGFR dependent manner. Flagellin, as well as the TLR2 ligand was also reported to stimulate airway epithelial migration, proliferation, survival, and wound repair [34]. Importantly, L94A functions similarly to the wild-type in cornel wound healing experiments. Thus, we conclude that activation of NF-κB, p38 and JNK pathways and/or the production of proinflammatory cytokines may not be required for flagellin to promote wound healing, and EGFR signaling is an integrated part of epithelial innate defense apparatus.

Taken together, we provide evidence for potential uncoupling of the major epithelial proliferation/repair pathway (i.e., EGFR) from the inflammatory pathway (i.e., NF-κB). By controlling the pathways to be activated, flagellin can induce an autonomous compensatory program at the ocular surface, resulting in increased production of antimicrobial peptides, cell migration, proliferation, and wound repair without cytokine production and tissue damage. Thus, flagellin and its derivatives may have broad therapeutic applications for cytoprotection and to control of infection in the cornea and other mucosal tissues surfaces.
